# Automated Movement Correction for Dynamic PET/CT Images: Evaluation with Phantom and Patient Data

**DOI:** 10.1371/journal.pone.0103745

**Published:** 2014-08-11

**Authors:** Hu Ye, Koon-Pong Wong, Mirwais Wardak, Magnus Dahlbom, Vladimir Kepe, Jorge R. Barrio, Linda D. Nelson, Gary W. Small, Sung-Cheng Huang

**Affiliations:** 1 Molecular and Medical Pharmacology, David Geffen School of Medicine at UCLA, Los Angeles, California, United States of America; 2 Psychiatry and Biobehavioral Sciences, David Geffen School of Medicine at UCLA, Los Angeles, California, United States of America; 3 Biomathematics, David Geffen School of Medicine at UCLA, Los Angeles, California, United States of America; Banner Alzheimer's Institute, United States of America

## Abstract

Head movement during a dynamic brain PET/CT imaging results in mismatch between CT and dynamic PET images. It can cause artifacts in CT-based attenuation corrected PET images, thus affecting both the qualitative and quantitative aspects of the dynamic PET images and the derived parametric images. In this study, we developed an automated retrospective image-based movement correction (MC) procedure. The MC method first registered the CT image to each dynamic PET frames, then re-reconstructed the PET frames with CT-based attenuation correction, and finally re-aligned all the PET frames to the same position. We evaluated the MC method's performance on the Hoffman phantom and dynamic FDDNP and FDG PET/CT images of patients with neurodegenerative disease or with poor compliance. Dynamic FDDNP PET/CT images (65 min) were obtained from 12 patients and dynamic FDG PET/CT images (60 min) were obtained from 6 patients. Logan analysis with cerebellum as the reference region was used to generate regional distribution volume ratio (DVR) for FDDNP scan before and after MC. For FDG studies, the image derived input function was used to generate parametric image of FDG uptake constant (K_i_) before and after MC. Phantom study showed high accuracy of registration between PET and CT and improved PET images after MC. In patient study, head movement was observed in all subjects, especially in late PET frames with an average displacement of 6.92 mm. The z-direction translation (average maximum = 5.32 mm) and x-axis rotation (average maximum = 5.19 degrees) occurred most frequently. Image artifacts were significantly diminished after MC. There were significant differences (P<0.05) in the FDDNP DVR and FDG Ki values in the parietal and temporal regions after MC. In conclusion, MC applied to dynamic brain FDDNP and FDG PET/CT scans could improve the qualitative and quantitative aspects of images of both tracers.

## Introduction

PET/CT is widely used in the clinic for oncological, neurologic and cardiologic applications. The CT image of PET/CT study not only provides anatomical information but also provides a means for scatter correction [1] and attenuation correction [2] to replace the transmission scan in PET-only scanners. Typical dynamic PET scans with molecular imaging probes usually last for one hour or more. Head movement is frequently observed during this time. Even for patients with belt restraint on the head, significant movement is obvious in late time frames. The problem is especially serious for elderly patients and patients with movement disorders.

The effects of head movements on attenuation correction and scatter correction in stand-alone PET scans have been previously investigated [3], [4]. Generally, head movements during dynamic PET/CT scans may create artifacts in attenuation correction and scatter correction due to the mismatch between the CT and PET images. The artifacts such as uneven tracer distribution cannot be eliminated by simply realigning the PET images in different dynamic frames to a reference frame or to the CT image alone [3]. Moreover, time activity curves (TACs) of different regions are usually extracted from dynamic images and are then used for kinetic modeling to calculate physiologic parameters such as uptake constant (K_i_) for FDG scans and distribution volume ratio (DVR) for FDDNP scans [5], [6]. Errors in the PET images may create incorrect physiologic parameters [7], [8].

Considerable work has been conducted to reduce motion artifacts on PET-only scanners. Two general approaches – by motion tracking system [9], [10] or with image-based realignment [3], [11], [12] – have been investigated to realign the transmission image to each emission frame for more accurate attenuation and scatter correction. Movement correction (MC) based on motion tracking system such as the Polaris system has been shown to be very accurate for phantom scans. However, due to fixation issues in human scans, image-based movement correction was found to outperform the other approaches in a majority of situations [9].

The aim of this study was to enhance both the qualitative and quantitative aspects of a dynamic PET/CT imaging study using an automated retrospective image-based MC method. This MC method not only corrects for movements between the CT image and each of the dynamic PET frames but also corrects for movements among the dynamic PET frames. Two tracers representing high and moderate signal-to-noise ratios, namely 2-[^18^F]-fluorodeoxy-D-glucose (FDG) and 2-(1-{6-[(2-[^18^F]fluoroethyl)(methyl)amino]-2-naphthyl}-ethylidene)malononitrile (FDDNP), were used to validate and evaluate the proposed MC method with phantom experiments and research patient studies. FDG, an ^18^F-labeled glucose analog, is most commonly used in nuclear medicine clinic to study brain glucose metabolism in neurodegenerative diseases [13]. FDDNP is a hydrophobic molecular probe used for *in vivo* imaging of β-amyloid senile plaques and neurofibrillary tangles – the neuropathologic hallmarks typically found in patients with Alzheimer's disease (AD) [14], mild cognitive impairment (MCI), Down syndrome (DS) [15], and progressive supranuclear palsy (PSP). Using those two tracers, the influence of head movement on tissue TACs was addressed and the effects of movement correction in parametric images of DVR for FDDNP scans and of K_i_ for FDG scans were also evaluated.

## Materials and Methods

### Movement Correction Procedure

**Figure 1:**
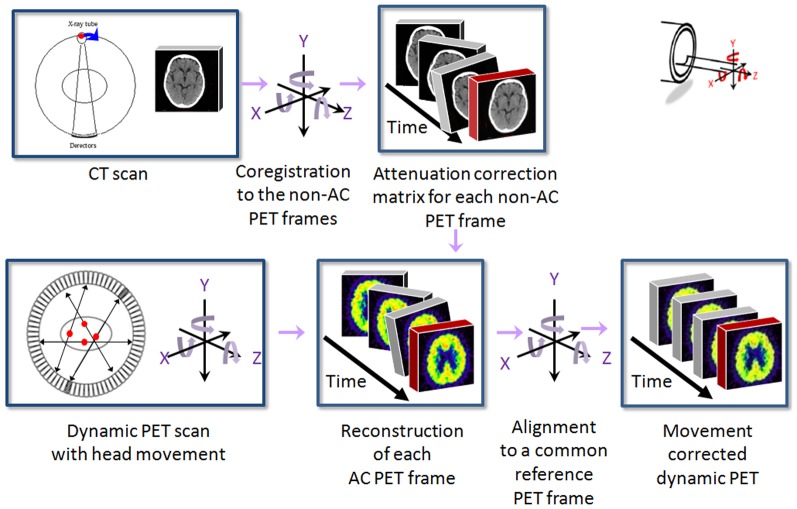
Illustrated overview of the retrospective MC method for dynamic PET/CT dynamic scan.

The automated retrospective MC method consisted of two major parts (Fig. 1). The first part co-registered the CT image to each of the PET frame in order to correctly provide attenuation information for the second part. The second part then re-reconstructed attenuation-corrected (AC) PET frames and aligned each frame to a common reference frame. The whole procedure was streamlined and automated using an in-house python script (ActiveState Software Inc. Vancouver, BC, Canada).

- Step 0: The CT and all PET frames of a dynamic PET/CT scan were reconstructed from raw data. A PET frame was selected to serve as a reference frame, which has a relatively high signal-to-noise ratio and shows clear anatomical features. In this study, the 1st, 7th, and 24th frames were chosen as the reference frames for phantom experiment, dynamic FDDNP scan, and dynamic FDG scan, respectively. For the reference frame, both AC and non-AC PET images were reconstructed; for other frames, attenuation correction was not applied.

Part I

- Step 1: The head holder was also in the field of view and contributed to attenuation. During a dynamic PET/CT scan, the patient may not be able to keep still and thus the patient's head might move but the head holder remained stationary. Therefore, only the patient head in the CT image needs movement correction. The first step of the MC method was to segment out the patient's head from the background in the CT image. This was done automatically by defining a contour around the patient head and setting the background outside the contour as −1000 HU (CT value of air) with an in-house MATLAB (MathWorks Inc.) program.
- Step 2: The original patient's head CT image (CT_ORIGINAL_) generated in the previous step was then co-registered (as a rigid body) to an AC PET image of the reference frame to produce a reference CT image (CT_REF_), which was aligned to the reference PET frame and contained no stationary part (i.e., the background structure such as the head holder).
- Step 3: The non-AC reference PET frame was then individually co-registered to all other non-reference non-AC PET frames to derive (n-1) transformation matrices, where n was the total number of frames in the dynamic scans. In FDG study, the short frames within the first 90 s were summed for a single co-registration, and the derived transformation matrix was replicated for each frame in the first 90 s and totally (n-1) matrices were still generated.
- Step 4: CT_REF_ image was then moved (n-1) times with the (n-1) transformation matrices obtained from Step 3. Thus, Step 4 produced (n-1) head CT images which were in the same position as the (n-1) PET frames respectively and those images had no stationary background.
- Step 5: The background (removed in Step 1), which contains stationary components, was added back to each of the moved CT images obtained in Step 4 automatically with an in-house MATLAB program.

Part II

- Step 6: Each AC PET frame was re-constructed with attenuation weighted-ordered subsets expectation maximization (AW-OSEM, 6 iterations and 16 subsets) using the correctly co-registered CT generated in Step 5 for attenuation correction.
- Step 7: Reconstructed AC PET frames were realigned to the reference PET frame using the transformation matrices derived in Step 3.

The co-registration of CT to PET used an algorithm that maximized the normalized mutual information, while the co-registration between PET frames maximized the normalized correlation coefficient. All image co-registration, realignment, and moving procedures were performed using Vinci software (Max Planck Institute for Neurological Research) [16].

### Phantom Studies

The co-registration algorithm and automated retrospective MC method were first evaluated and validated on phantom studies. A Hoffman Phantom filled with FDG (∼1100 ml in volume and ∼13 MBq when the scan started) was scanned by a Biograph 64 TruePoint PET/CT scanner (Siemens, Inc.), which has an intrinsic resolution of approximately 4.5 mm full width at half maximum (FWHM). To validate the accuracy of the co-registration algorithm used in the MC method, a computer controlled motion platform was used to conduct pre-set translational movements of the Hoffman Phantom in different PET frames, and the images were aligned to determine the amount of movements, which were then compared with the pre-set values. Twelve static PET frames with 10 minutes each were acquired at different positions with the first frame as a reference frame. One CT image (CT1) was acquired with the phantom at the same position as the first PET frame (FR1) and another CT image (CT12) was acquired with the phantom at the position of the last PET frame (FR12). In the phantom studies, AC and non-AC PET images were both reconstructed.

Table 1 shows the relative positions of the phantom during the 12 frames, in reference to that of FR1. The Displacement of one frame to the reference frame was defined in Equation 1.
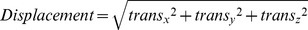
(1)The accuracy of the co-registration was evaluated by the error between the pre-set displacement and the displacement calculated from the resulted co-registration parameters. The error is defined in Equation 2.
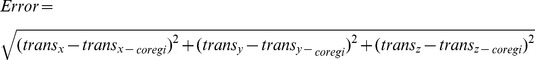
(2)The MC procedure (described in the section of “Movement Correction Procedure”.) was evaluated by examining the differences among PET images that were attenuation corrected with different datasets– aligned, mismatched and movement corrected CT images. It should be noted that the measures of Displacement and Error defined above are only for the location at the center of the image. Without rotational movements (like in the phantom experiment with platform movements), the displacement measures are also applicable to everywhere in the head. However, if there are rotational changes, the actual amounts of location movements in most regions of the head are much larger than indicated by these measures.

**Table 1:** Relative positions of the Hoffman brain phantom for different CT scans and PET frames.

	CT1	Dynamic PET Scan												CT12
		FR1	FR2	FR3	FR4	FR5	FR6	FR7	FR8	FR9	FR10	FR11	FR12	
Y*(mm)	0	0	0	−1	0	2	0	5	5	−10	−18.9	−2	10	10
Z#(mm)	0	0	1	0	−2	0	−5	0	10	20	0	2	−10	−10

*: Y is the direction perpendicular to the bed panel.

# : Z is the direction parallel to the long axis of bed.

The positions were in reference to that of FR1.

In another phantom study, the MC procedure was further evaluated by incorporating various translational and rotational movements as well as their combinations into different dynamic scanning frames to simulate head movements at different times. The resultant images were evaluated in terms of artifacts and the amount of quantitative errors in image value.

### Patient studies

The automated retrospective MC method was applied to representative patient data and the effects of MC were evaluated. All patient studies were approved by the Human Subjects Protection Committee at UCLA, and written informed consents were obtained from all subjects. The patient datasets used included dynamic FDDNP and FDG PET/CT scans on DS, AD, MCI and PSP patients performed on the same scanner used in the phantom experiments. A curved head holder attached to the patient bed of the scanner was used to help minimize the head movement of the patients. Patients laid their head supinely on the head holder. The CT scan was acquired first, and was used for generating attenuation correction factors. Tracer (∼370 MBq) was injected intravenously as a bolus and a dynamic PET scan was initiated immediately thereafter. A moderate physical restraint through the use of a belt over the patient's head was applied during the entire scan of over 60 min.

#### Dynamic FDDNP PET/CT Scan Protocol

Dynamic FDDNP PET/CT images were obtained from 12 subjects (8 DS, 1 AD, 2 MCI and 1 PSP). FDDNP was synthesized using the method previously reported [17]. Total dynamic PET acquisition time was 65 min (6×30 s, 4×3 min, 10×5 min).

#### Dynamic FDG PET/CT Scan Protocol

Dynamic FDG PET/CT images were obtained from 6 subjects (6 DS). Total dynamic PET acquisition time was 60 min (9×5 s, 3×15 s, 3×30 s, 1×2 min, 5×5 min, 3×10 min).

### Quantification

To evaluate the effect of MC on the biological quantitation of the dynamic PET imaging in human studies, the following processing steps were applied to the dynamic images to extract the biological parameters. The same procedures were applied to dynamic images without MC and with MC, and the results were compared. The effects of MC were also examined in terms of artifacts and noise reduction.

#### Region-of-Interest (ROI) Analysis

ROIs were defined manually on the frontal cortex, lateral temporal lobe, medial temporal lobe, parietal cortex, posterior cingulate and cerebellum. Tracer TACs of these regions were obtained.

#### FDDNP Distribution Volume Ratio (DVR)

Volume of Distribution of tissue (V_T_) is defined as the ratio between tracer concentration in tissue (C_T_) and tracer concentration in plasma (C_P_) at steady state (Equation 3):
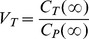
(3)Distribution volume ratio (DVR) is defined as ratio between V_T_ and volume of distribution in the reference region (V_REF_) of no specific binding (Equation 4):
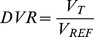
(4)Logan analysis [5] was used to estimate the DVR as the slope in the linear range of the Logan plot (Equation 5):
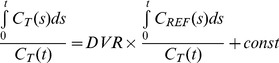
(5)where C_REF_(t) is the tracer concentration in cerebellum which was chosen as the reference tissue for FDDNP PET/CT studies. The Logan plot was considered linear 30 minutes after scan start time in dynamic FDDNP scan. DVR value of each pixel was estimated and DVR image were generated on the dynamic FDDNP scans (with and without MC).

#### FDG Uptake Constant (K_i_)

To calculate K_i_, image-derived input functions (IDIFs) (C_P_(t)) were obtained from the carotid artery (CA) ROIs [18], [19]. The early images over the first 45-second period were summed for defining CA ROI. The CA ROI consisted of 5 mm diameter circles manually defined on the CA in each of three consecutive slices that had clearly visible carotid artery below the temporal lobe. Parametric images of K_i_ were generated with Patlak analysis (Equation 6) [20] using TACs extracted from CA ROI as the IDIF.
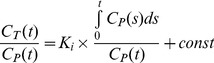
(6)Both IDIF and the K_i_ values from images with MC were compared to those without MC.

## Results

### Phantom Studies

**Figure 2:**
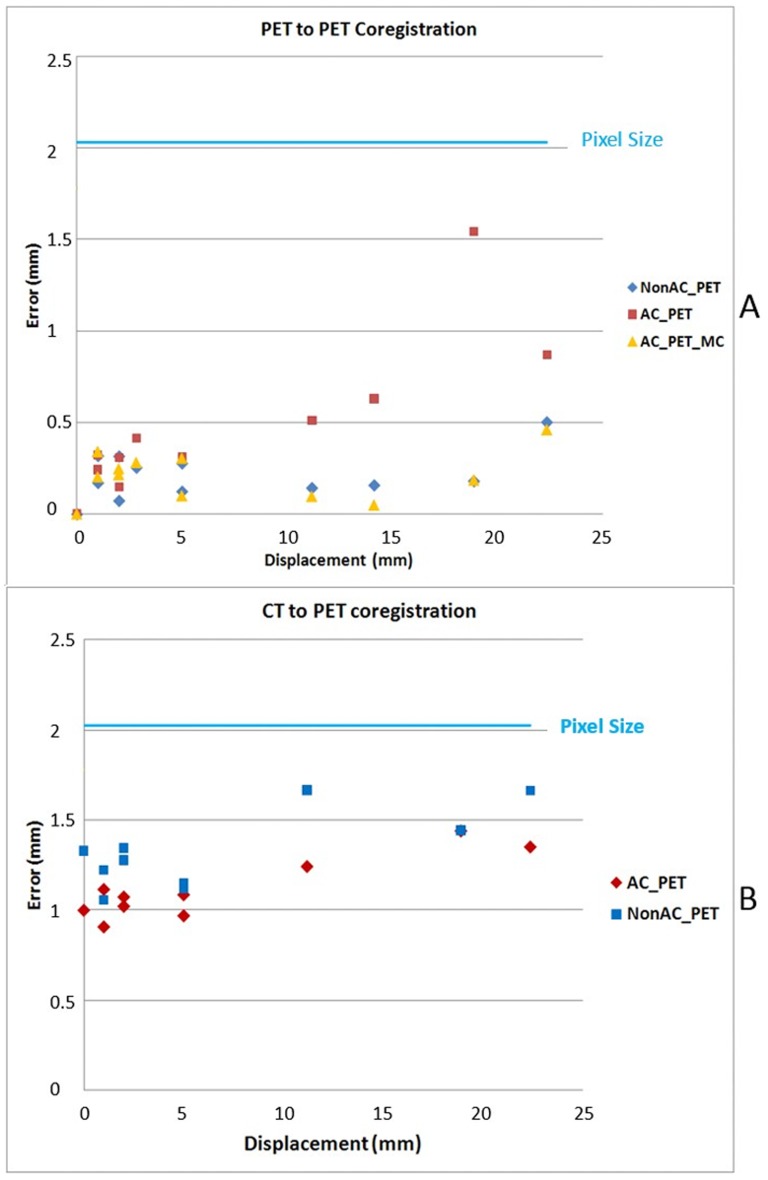
Co-registration accuracy evaluated from FDG phantom study. **A.** PET to PET co-registration accuracy **B.** CT to PET co-registration accuracy.

Co-registration errors for PET images of different AC procedures are shown in Fig. 2A. The errors of co-registration between PET images of different frames based on the PET images without AC was smaller than the errors based on the PET images with AC (before MC), but were similar to those based on the AC PET images after MC. The difference was contributed by attenuation correction artifacts. However, the co-registration error was always smaller than the voxel size (Fig. 2A). In contrast, the errors of CT-to-PET co-registration based on the PET images without AC were slightly larger than those based on the AC (before) PET images (Fig. 2B).

**Figure 3:**
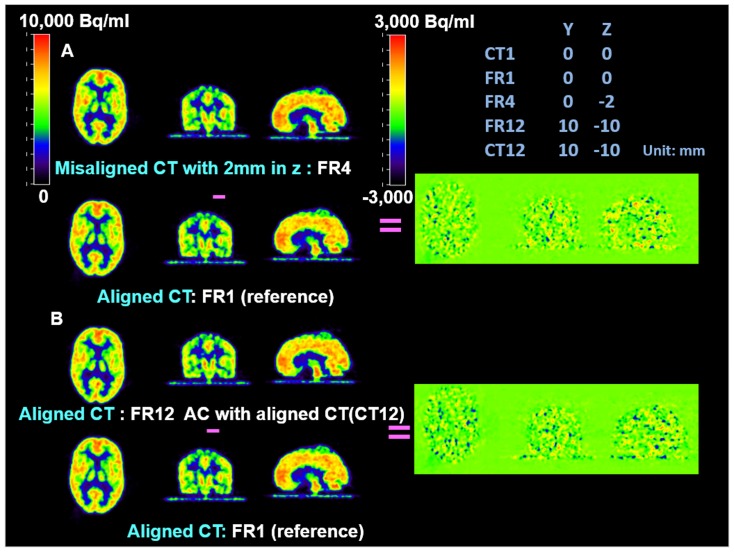
Comparison of AC with slightly misaligned CT and fully aligned CT in FDG phantom study. **Panel A:** the difference map between PET FR4 AC with a 2(CT1) and PET FR1 AC with an aligned CT (CT1) (taken as the reference PET frame). The small misalignment (2 mm) between PET and CT has subtle impact on AC comparing to Panel B.; **Panel B:** the difference map between PET FR12 AC with an aligned CT (CT12) and the reference. The difference map shows only the noise.

**Figure 4:**
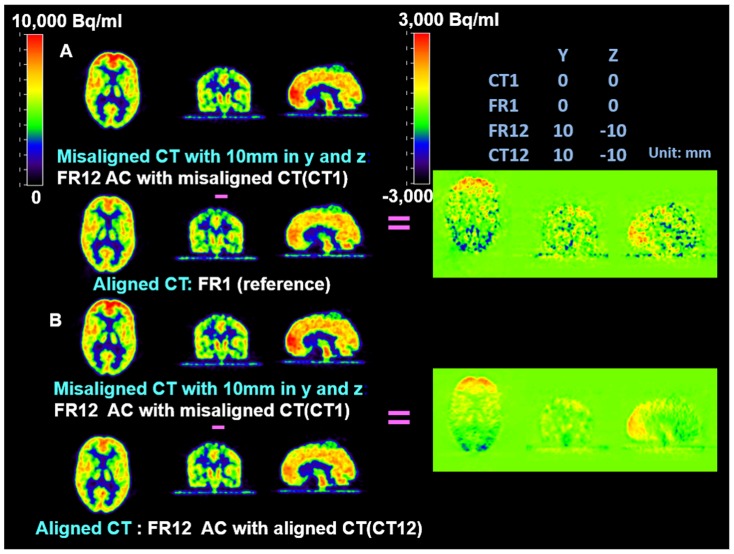
Artifacts of AC with largely misaligned CTs in FDG phantom study. **Panel A**: the difference map between PET FR12 AC with a 10 mm mismatched CT (CT1) and the reference PET image. The misalignment between PET and CT caused significant AC artifacts; **Panel B:** the difference map between PET Frame12 AC with a 10 mm mismatched CT (CT1) and the same PET frame AC using the physically aligned CT (CT12). The misalignment between PET and CT caused significant AC artifacts. However, the AC matrix did not add extra noise to the PET images.

**Figure 5:**
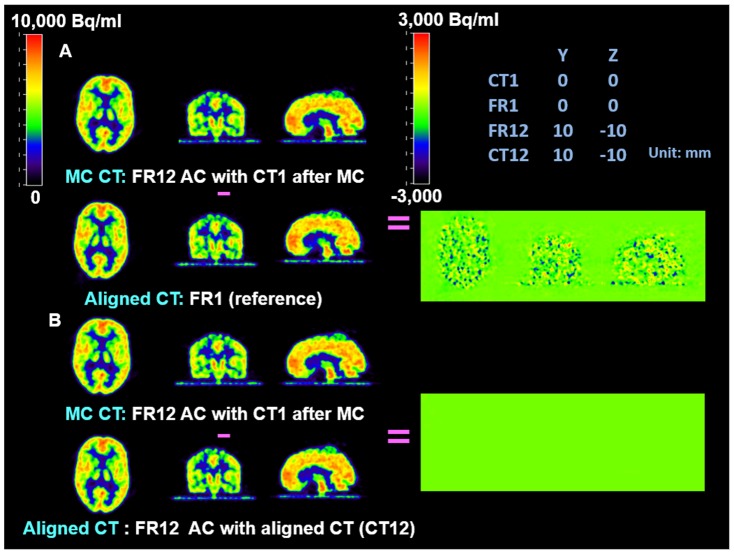
Comparison of AC with aligned CT and MC CT in FDG phantom study. **Panel A**: the difference map between PET FR12 AC with movement corrected CT1 and the reference image. After MC, there were no noticeable differences, except noise, among the PET frames; **Panel B:** the difference map between PET FR12 AC with movement corrected CT1 and the same PET frame with the correctly aligned CT (CT12).

Difference maps were used to further evaluate and validate the MC procedure on the phantom studies. They showed that the accuracy of co-registration (error <2 mm) was adequate to avoid introducing extra artifacts and the MC procedure eliminated artifacts due to large mismatches between PET and CT images. In Figs. 3, 4, and 5, all PET images were co-registered to the reference frame for comparison. The same color scale was applied across all PET frames. A different color scale was applied to the difference maps, where green denotes zero difference. Fig. 3 shows that for small misalignment (2 mm in z direction) between PET and CT, the AC artifact was not noticeable compared to the images with correctly aligned PET and CT. Fig. 4 shows apparent artifacts due to large mismatches between PET and CT images. Panel A of Fig. 4 shows the difference map between the PET frame (FR12) which was based on a misaligned CT (CT1) for AC and the reference frame (FR1) which used a physically aligned CT (CT1). The difference map between the FR12 and the same PET frame based on a physically aligned CT (CT12) is shown in Panel B. Fig. 4 shows that the misalignment between PET and CT caused significant AC artifacts. However, the attenuation correction matrix did not add extra noise to the PET images. After MC, there were no noticeable differences, except noise, among the PET frames (Fig. 5).

**Figure 6:**
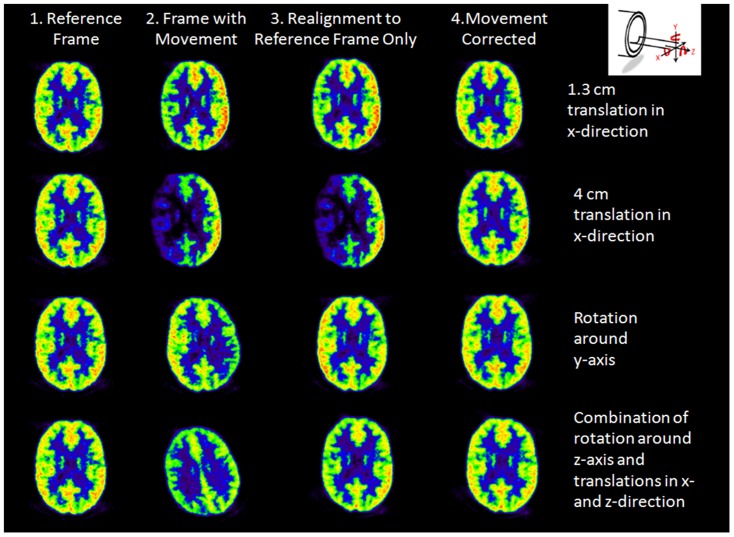
Movement correction for PET/CT misalignment induced by head translation and rotation in FDG phantom study.

Result of the application of the automated MC procedure to the second phantom study is shown in Fig. 6 that demonstrated the procedure's ability to correct misalignment induced by head rotation besides translation. Co-registration to the reference frame (without CT MC) (column 1) only reduced the attenuation artifact (column 3). Mean left-right asymmetry decreased significantly (P<0.05) from 46.8% to 6.5% after MC (column 4).

### Evaluation with Dynamic FDDNP PET/CT Scans

**Figure 7:**
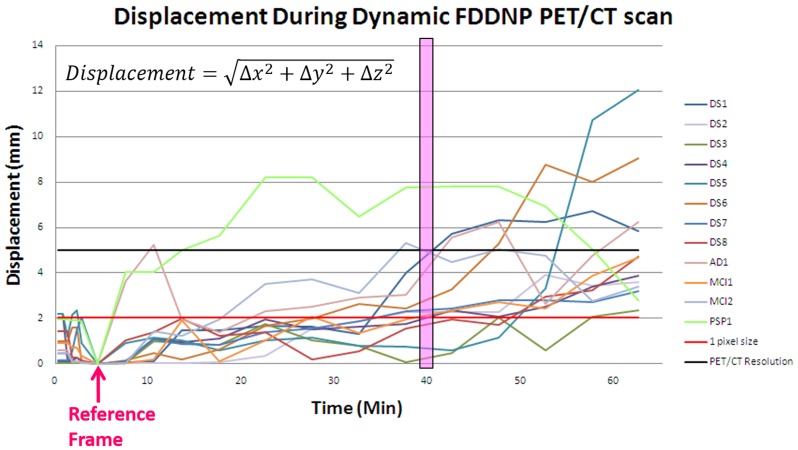
Displacement during dynamic FDDNP PET/CT scan. Half of the subjects have displacements over 5(after 40 min).

If the displacement was larger than 5 mm (resolution of the PET/CT imaging system) or the rotation was greater than 4 degrees, the movement would cause visually noticeable artifacts in the PET images. Fig. 7 shows that half of the subjects had displacements over 5 mm in late frames (after 40 min). The z-direction translation and nodding were found to be the most common patient movement during a dynamic PET/CT scan. Under-attenuation or over-attenuation correction artifacts were observed in frontal or parietal regions due to mismatch in the z direction.

**Figure 8:**
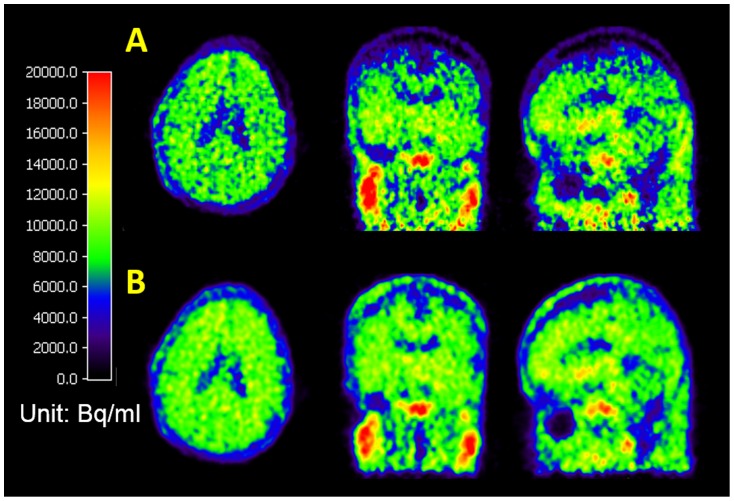
Improvement of a late FDDNP PET/CT frame image after MC. **Panel A:** An FDDNP PET/CT study on a Down syndrome patient with 10 mm movement in z direction in a late frame of a dynamic scan. **Panel B:** 3-D orthogonal images show the improvement of the PET/CT images with proper AC after MC. All images are displayed with the same color scale and show clearly the quantitative differences in the PET images reconstructed with misaligned and aligned CT images.

Fig. 8 shows the improvement of the PET/CT images with proper AC after the automated MC procedure in a dynamic FDDNP PET/CT scan on a DS patient. Panel A shows the FDDNP PET images reconstructed based on a CT image that was misaligned (due to head movement between CT and PET imaging). Panel B shows the same PET image attenuation corrected and reconstructed based on the CT image that was properly aligned after MC. Significant differences were seen between images in the top and bottom rows in the figure.

**Figure 9:**
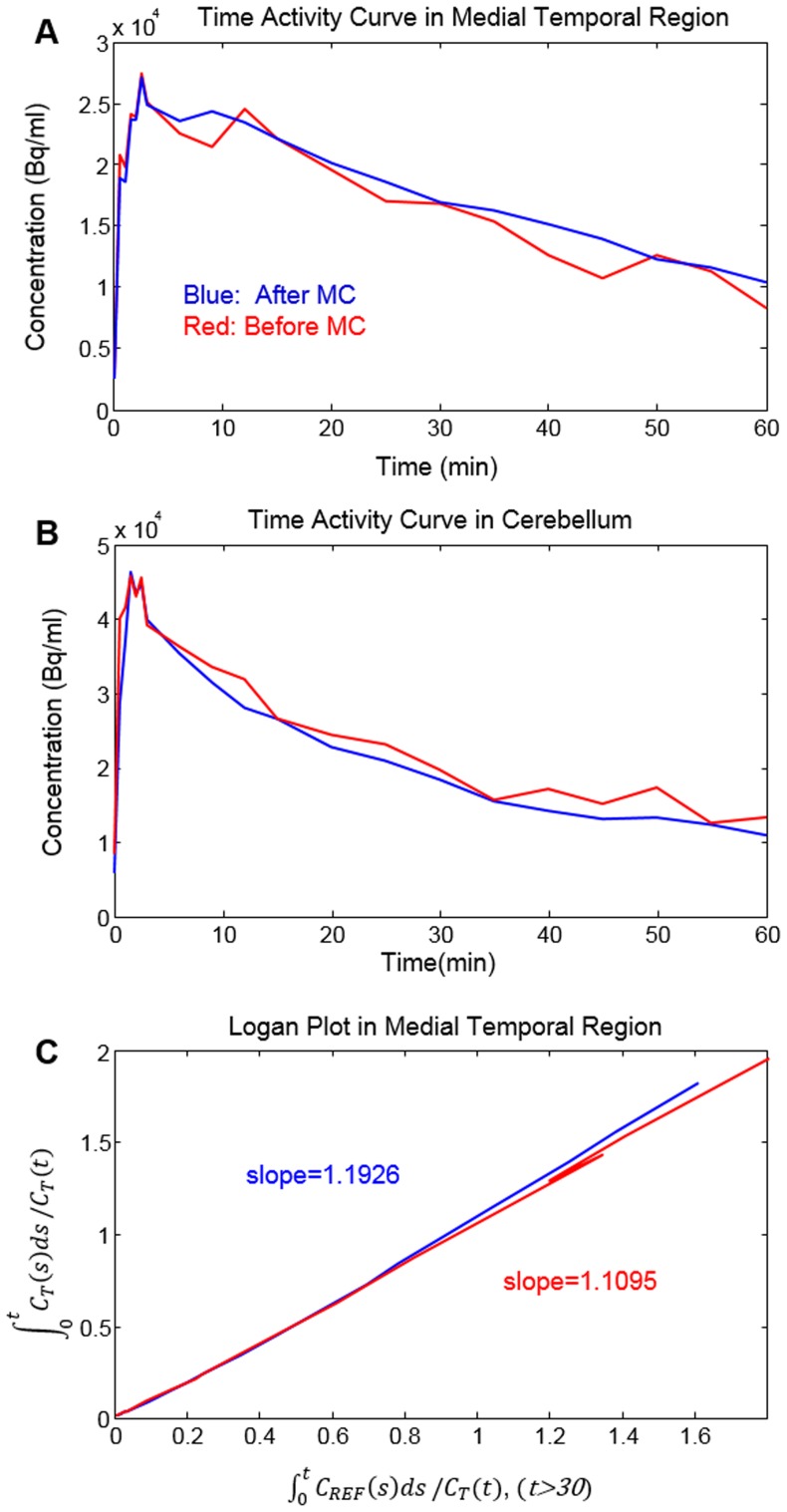
TACs in both medial temporal region and cerebellum (the reference region), and Logan plot showed less fluctuation after MC for a subject who had large head movement during a dynamic FDDNP PET/CT scan. **A**. Comparisons of TACs before and after MC in medial temporal region. **B**. Comparisons of TACs before and after MC in cerebellum. **C**. The slope of the Logan plot increased by 7.5% after MC for this patient.

**Figure 10:**
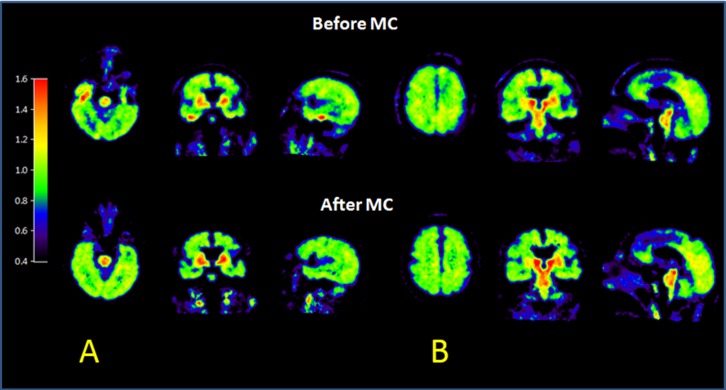
A FDDNP PET/CT scan subject with both head nodding and shaking movement showed reduction in artifacts in the temporal region, reduction in left-right asymmetry and better hemisphere separation after MC. **Panels A and B** show different brain slices of the same subject before and after MC.

**Figure 11:**
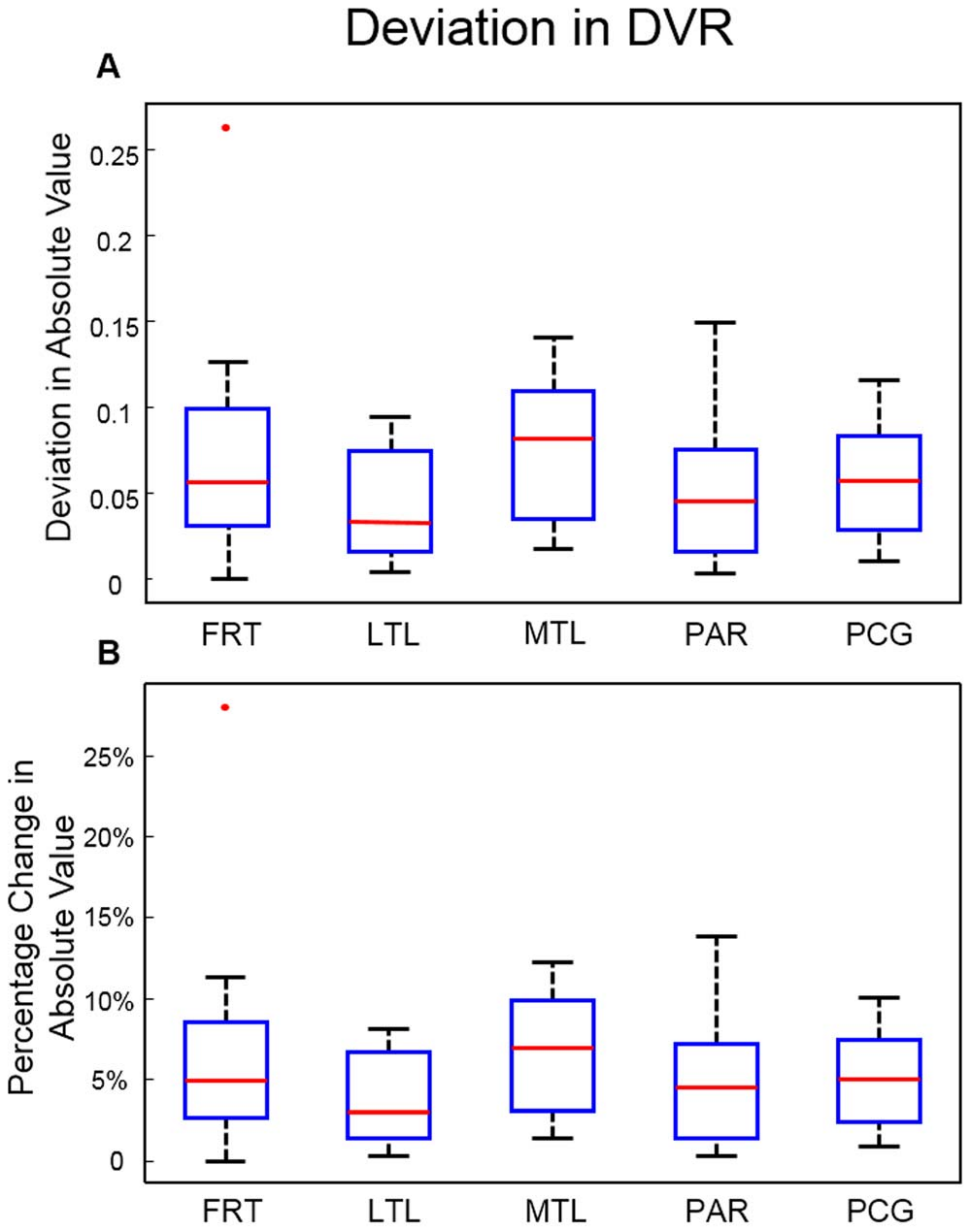
Boxplot showing difference of DVR in Regions of interest (ROIs) before and after MC. **A.** in absolute value and **B.** in percentage of absolute value. ROIs include frontal cortex (FRT), lateral temporal lobe (LTL), medial temporal lobe (MTL), parietal cortex (PAR) and posterior cingulate (PCG).

Since the tissue TAC of FDDNP in the medial temporal region usually becomes linear later in time when plotted on a Logan plot, we used it to determine the time range which is 30–65 minutes for DVR image generation. Comparison of TACs before and after MC and their corresponding Logan plots for a medial temporal region in a patient is shown in Fig. 9. The TACs in both medial temporal region and cerebellum (the reference region), and Logan plot showed less fluctuation after MC. The slope of the Logan plot increased by 7.5% after MC in the medial temporal region for this patient. Improvement in the DVR image after MC is shown in Fig. 10A for a patient who had head rotations and translations. Reduction in artifacts in the medial temporal region, and in left-right asymmetry after MC was easily noticeable. The same study also showed better hemispheric separation, increased uptake in brain stem and occipital regions, and decreased uptake in frontal region after MC (Fig. 10B). In the FDDNP research patient study group, DVR values in the frontal lobe and medial temporal region were significantly different (P<0.05) before and after MC; the absolute value of difference of DVR before and after MC was 0.074 (6.9%) and 0.076 (6.6%), respectively (Fig. 11).

### Evaluation with dynamic FDG PET/CT scans

**Figure 12:**
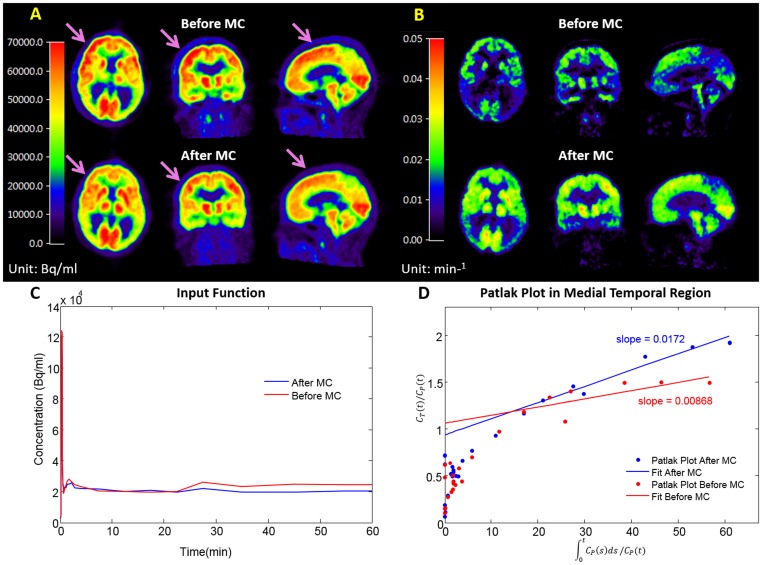
Improvement of a FDG PET/CT scan of a DS patient after MC in aspects of late frame, K_i_ image, image derived input function and Patlak Plot. **A.** Orthogonal PET/CT images before and after MC of a late frame in the dynamic brain FDG scan: attenuation correction artifact in the right frontal region (pointed at by arrows) diminished after MC (lower row). **B.** The K_i_ image: The K_i_ values appeared to be more uniform (except the affected regions) and globally higher after MC. In this case, the lower input function accounted for the global increase in the K_i_ values **C.** Image Derived Input Function: After MC, a 21% drop in the tail AUC during 30–65 minutes was seen. **D.** Patlak Plot in the medial temporal region before and after MC: The Patlak plot had less fluctuation after MC. The slope of the plot used for calculating K_i_ increased by 97.9% after MC for this patient.

Movements were observed in all studied subjects in late PET frames with an averaged central displacement of 5.55±2.9 mm. Artifacts were observed in late dynamic frames, in the K_i_ image, in the IDIF and in the Patlak plots before MC, and were significantly reduced after MC, as shown in Fig. 12 for a representative patient study. The total area under the curve (AUC) and the AUC between 30 and 60 minutes post-injection of FDG (tail AUC) of the IDIF obtained from CA ROIs were respectively changed by 33% and 57% on average after MC. The K_i_ values were changed after MC in posterior cingulate, frontal, parietal, and temporal regions by 44.6%, 46.4%, 53.2%, and 48.3% on average respectively. The coefficient of variation of K_i_ value among the 6 subjects over those regions decreased from 68.4% to 20.9% after MC.

## Discussion

In this study, a retrospective image-based MC method for a PET/CT scanner has been developed and validated. The MC method for dynamic PET/CT scan has been shown to be practical and easy to use. The MC method requires the co-registration of medical images obtained from different imaging modalities and from the same modality. The co-registration for both inter- and intra-modality has been under intensive research during the past fifteen years. Maximization of mutual information has been shown to be an excellent criterion for automated and accurate rigid-body co-registrations of intra-individual images from different modalities especially between PET and MRI in a variety of applications [21]. Multiple software packages (e.g., Vinci [18] and SPM [22]) use this criterion for medical image co-registration. In our implementation of the MC method for PET/CT imaging, we have adopted the use of Vinci for image co-registration, since the pre-processing procedures available in Vinci, including thresholding, sped up the processing and provided robust co-registration [23].

The 3-D Hoffman brain phantom is commonly used to simulate cerebral blood flow and metabolic PET images. It not only mimics the tracer distribution but also simulates the photon attenuation and scatters in the acquired images [24]. The Hoffman brain phantom was used in this study to demonstrate that the algorithms implemented by Vinci was adequate for cross-modality co-registration between PET and CT and for between frame PET co-registration (Fig. 2). For co-registration between PET and CT, the PET images with AC outperformed those without AC, even though there might be artifacts in PET with inaccurate AC based on mismatched CTs. This could be due to the reason that there was a stronger signal inside the brain for PET image with AC. We found in the present study that the co-registration almost reached half-pixel accuracy, which is consistent with results reported by others [25]. In contrast, for co-registration between PET frames, the use of PET images without AC outperforms the use of PET images with AC. It is worth mentioning that, for PET images with AC after MC, the co-registration accuracy reached the same level as that for PET images without AC. This result indicates that the artifacts in AC influence the co-registration accuracy among PET frames. Thus we optimize the MC procedure using AC PET for co-registration between CT and reference PET frame and non-AC PET for co-registration among PET frames.

Multiple static Hoffman phantom scans only mimic FDG distribution in the brain at pre-defined time points after tracer injection and do not exactly reflect the actual FDG dynamics in patient's brain, in which the radioactivity distribution changes continuously over time (Table 1 and Fig. 6). The evaluation of the MC method was thus further performed on patient dataset of dynamic FDDNP and FDG PET/CT scans (Figs. 7, 8, 9, 10, 11, 12). The accuracy of co-registration in patient is hard to quantify because the true movement was not recorded in our study. However, visual check of the co-registration showed that the co-registration algorithm works well on early frame as well. Though the pattern of the tracer distribution might be different in early times among different frame, the image patterns in early frames of FDG and FDDNP dynamic scans did not change much and did not affect the co-registration of early frame images. To help with the co-registration for early frames which have low counting statistics in FDG study, those short frames in the first 90 seconds were summed for co-registration between PET and CT. Figure 3 shows that small error in co-registration between PET frame and CT image could be tolerated in attenuation correction. On the other hand, as shown in Fig. 7, with some physical restraint, movement in early frames could be well limited. The MC was more important for Logan and Patlak analyses than for compartment fitting because the slope in both Logan and Patlak plots are mainly determined by the late phase of tracer uptake. However, if the scan durations of the early frames were very short or the tracer used has a low uptake at early time, the high statistical noise could cause problems for image alignment and would affect the results of compartment modeling.

Both types of error (due to the image derived input function and the tissue concentration kinetics) could contribute to the change in K_i_. Similarly, both types of error (due to the reference region and the tissue concentration kinetics) could contribute to the change in DVR. The relative importance of the two error types is highly dependent on the tracer used, the reference region available, and the quantitation methods employed. For example, the carotid artery ROI used for deriving the FDG input function had a much smaller size than the cerebellum used for FDDNP PET scans as the reference tissue and was affected more by movement; these explain why the impact of movement correction on the FDG results was much higher than on the FDDNP results. The relative effects of the two types of error on FDDNP and FDG quantitation are stated respectively in the legend of Fig. 9 and Fig. 12.

In our MC evaluations with patient images, the effect of head movement and MC could not be done in absolute terms. For example, in the evaluation of their effects on the FDG K_i_ values, the exact percent changes should be interpreted with caution. Since no spillover and partial volume correction was applied in estimating the blood TACs, the input functions used in calculating the K_i_ values were not exactly correct. In addition, noise in C_T_(t) measured from PET imaging could contribute to negative bias in DVR estimates obtained by graphical methods and this underestimation is noise level dependent [26], [27]. However, we believe that the results shown in Figs. 10 and 12 do represent the potentially large effects of head movement on the FDDNP DVR and FDG K_i_ values that can be significantly reduced with the MC method presented in this study.

Additional smoothing might be introduced through the MC procedure. In practice, to avoid this smoothing in the case of negligible head movement, a general check of the magnitude of the movement based on the transformation matrices is performed. When the amount of head movement is small, the user has the option to use the original images (i.e., without MC) instead of the ones with MC (which have a small amount of additional smoothing).

For the patients who had neurodegenerative disease or could not remain stationary during a dynamic scan, even with a shortened dynamic scan (e.g. from two hours [3] to one hour) and with the use of a head belt for restraint, head movements were clearly noticeable in half of the subjects studied (Fig. 7). Common head movements observed in this study include translation in the z-direction, rotation around the z-axis (left-right head rotation) and rotation around x axis (head nodding). With the back of the head as a pivoting point, forehead generally had a larger displacement compared to the back of the head, which is consistent with our previous report [3]. Therefore, the frontal, parietal and temporal cortices that are important for AD evaluation [28], [29] are particularly sensitive to head movements during dynamic PET scans (Figs. 6, 8, and 9), and the automated MC method reported in this study is expected to improve the most for the dynamic PET/CT image results from these types of patients.

## Conclusion

The retrospective image-based MC method described in this study is feasible for dynamic FDDNP and FDG PET/CT brain scans. It significantly improves the image quality and the measured tracer kinetics derived from dynamic PET/CT images. The MC thus should be applied if reliable DVR and K_i_ estimations on neurodegenerative patients are desired. The proposed MC method is also expected to be applicable to PET studies of patients with other disorders (e.g., Parkinson's disease) and to brain PET scans with other molecular imaging probes, but separate validation studies should be performed for each case.
